# Filling Teeth

**Published:** 1851-01

**Authors:** James Taylor


### ARTICLE XIII.

### *Filling Teeth.* By JAMES TAYLOR, M. D., D. D. S.

THE cavity having been prepared for a filling, by the entire removal of the disease, the margin being firm, smooth, and somewhat regular, that is, not notched and ragged, or the edge frail and brittle. The walls extending inward, being as near perpendicular as possible, or if beveling, so as to slightly enlarge the cavity within, yet not so as to form a neck, but the wall to present a continuous plane, so that a fold of the gold may rest against it smooth and straight its entire length.

Let me here draw attention to one circumstance, which, if neglected, will ultimately show itself around the margin of the filling. It is this—that in shaping these cavities, that is, in removing the carious portion, if particular care is not taken, the diseased bone just beneath the enamel will not be removed, and we will have a neck shaped cavity, the neck of which will be filled by caries. The decay will generally be found full as large at the entrance of the cavity, immediately under the enamel, as within. A portion of the enamel yet apparently unaffected by the disease, laps over the decay; this, in the brown and white variety alluded to, may be still unchanged in color; yet, with a proper shaped excavator, which can be thrown in under the enamel, it will be found soft, or undergoing decomposition. When this is ascertained to be the case, it shows that the enamel has not been sufficiently removed. This diseased bone I generally remove first, and then trim the border to suit; or, as is often the case, cut and trim until I find this part of the cavity perfected. Just so in the use of the drill; the first one used cannot, and generally should not, be as large as the decay, but after using one that will pass in readily, cut the decay from under the enamel; then a larger drill, and repeat this till this part of the cavity is perfected. As I before remarked, however, the first step in the preparation of the cavity, is the breaking down of the enamel which covers the decay, with the cutting instruments, and this is done to the extent necessary thoroughly to expose the disease.

We pass on to the next step in this operation, and this would be the preparation of the gold; for this should be prepared and ready for insertion before the cavity is washed out and dried. I am aware that various plans are adopted to accomplish this object. Some cut the gold in strips of about one-fourth of a sheet; then roll into a cylindrical form, adding strip after strip to this roll, till enough is added to fill the cavity; then make a few folds at one end, sufficiently large to be retained in the cavity when put in, till other folds are made with the plugger and forced upon it, filling up, by these repeated folds, the entire cavity. Others take two or three sheets of gold, lay carefully together, then fold once or twice, so as to get some ten or twelve thicknesses of the foil, then cut in strips as wide as the cavity, leaving these strips all united; then roll up one end into a ball sufficiently large to be retained in the cavity; then fold in, in regular and successive layers, as much as can be forced into the cavity. This plan, when done by an expert operator, goes far to the accomplishment of all that is desired.

Some difficulties, however, present in the proper introduction of the gold in this manner. Let us take, for instance, a central cavity. You commence with a strip, introducing the folded end first, then with your instrument draw up and force in another strip; let this be done with all the care imaginable, and all these strips will not be of one length; some will have been drawn entirely within the cavity, while others will project without. A few *misfolds* of this kind will occasion two things: first, the too rapid filling up of the cavity within for the border; and second, an irregularly compacted surface for the filling, rendering it more difficult to put on a fine finish. The same objections hold good as regards the first plan.

But this condition of things is far preferable to that which often takes place. The first fold or ball is forced into the bottom of the cavity, the next on top of this, and so on, filling up from the bottom to the top. If this plan is adopted, and it certainly is the easier, so far as the filling up of the cavity is concerned, yet what is to hold the last fold of gold. True, this may be worked into (more or less) that which is beneath, yet, if I mistake not, such fillings will generally be found to wear down pretty fast, and be far easier picked out than when the folds are compacted in the other way. Others again roll up small balls of gold, and force in one after another, beginning at the bottom of the cavity. The great desideratum undoubtedly is, to fill up the entire cavity, make the gold as solid as possible, impervious to air or moisture, and so compacted and held together, that one portion cannot be removed without the whole, unless by the aid of a cutting instrument.

That plan which will secure the introduction of the most gold, and best fill up the entire cavity, is certainly the best, and that which experience and long practice has made easiest to one, may be, for the want of this, almost impossible to another. Well do I remember the first filling I attempted to introduce with my foil cut into strips, and with foil such as recommended by one of our best dentists. This foil was rather hard yet adhesive, so that when folded it would stick together; when once introduced, it made a hard plug, and bore a fine polish; yet, after long and tedious labor, I tore my plug entirely out, and took up my cylindrical rolls. This to me then was *the* plan. But one failure did not discourage me; I tried other foil, and at length nearly threw my rolls aside. Notwithstanding I did this, and preferred my gold in strips, yet the objections already alluded to, determined me to try another plan, and I commenced this in the central cavities, for I found these, especially in the second molars superior, also inferior, to be as difficult to fill neatly as any other, which was anything like as accessible. I commenced by taking my strips, such as I had been using; these I cut, say one-third or fourth longer than the depth of my cavity. I then carefully rolled these into blocks of various sizes, having one sufficiently large to retain its position in the cavity when introduced. The object being to place these perpendicularly in the cavity, and fill up from the farthest portion of the cavity forward; but to do this the ordinary plugging instruments would not answer. For a long time I used a couple of pair of pliers, so shaped at the point as to resemble the ordinary sharp pointed plugging instruments used for these cavities.

I have now perfected what I call a set of plugging pliers or forceps, consisting of five pair, to these I shall probably add one or two more. The five are made from patterns of instruments most used in general filling. When closed, they resemble at the point these instruments, being sharp at the point, and so bent as to enter almost any cavity accessible with any instrument. When closed, therefore, they can be used as the ordinary instrument; and are thus often used for compacting the gold as introduced; and so admirably do they answer for this purpose, that in using the strips for filling, I use also these forceps. They possess one advantage over the ordinary plugger, which is, that with them you can catch hold of the strip at any point desired, carry it direct to the bottom of the cavity, and thus secure the desired length of fold. The spring of these instruments is such as to permit them to be closed with slight pressure, yet such as to force the blades some half inch apart, when this pressure is entirely removed. They are about five or six inches long, this should be determined by the length of hand, &c. Let us now take as an illustration of the method of using these instruments, the filling of a central cavity in an inferior molar. The cavity has been prepared, a napkin is held in place around the tooth with the speculum. The cavity has been thoroughly dried by the use of cotton, tissue paper, lint, or some such substance, which will most readily take up the moisture. The foil has been rolled up in blocks to suit the size of the cavity requiring some four, six, eight or ten, to fill the cavity depending on its size. These blocks you have laid perfectly within your reach. You then take a pair of forceps bent at near right angles; with these take first your largest block, place this on end in the posterior part of the cavity, having the cut border of gold projecting out of the cavity, say one-fourth the depth of the cavity; the other end, of course, resting on the bottom. When this block is to its place, and forced back solid against the posterior wall of the cavity, another next in size is introduced in like manner, and forced back against this; but it must here be remembered that this block will not, as the first, reach across the cavity; hence it should be forced to one side. The third block to the other, the fourth to the center, and so on until the cavity is completely filled. As each block is introduced it is pressed solidly back to its place, the last block introduced has a portion of the strip unrolled, and as much of this as *can be*, is forced in as a closing wedge. We here see the propriety of having the walls of the cavity perpendicular, so that these blocks will rest solid against them without being bent or broken down. In large cavities, after the introduction of each, or two or three blocks, a strong plugging instrument is used to force back and down these blocks, and thus give room for the introduction of others. We have here a filling made up of continuous layers of gold, each layer projecting above the cavity. No fold too long or too short; for in deep and large cavities the bottom can and should be first filled up to a level with the shallowest part of the cavity, by the introduction of one or more blocks laid in on their sides, and made perfectly solid. And this I do in all those cavities of much size, and where there is much tenderness at the bottom. A little experience will soon direct the most proper size and firmness of the blocks to be used, also their length. I have found that if properly introduced, and consolidated as introduced, that but little pressure on their ends is necessary to finish the compacting of the gold; yet this part of the operation is very important, as pressure should be made as the tooth will well bear. For this part of the operation a few strong instruments are necesary. These I have constructed in a manner which render them fit only for this purpose. I take the ordinary straight, curved, and flat compressing instruments, but the points are cut like a file, the sides of the flat are also made this way. These are to be used after the introduction of the gold. Two objects are had in view in having my compressing instruments thus made. The first is, they can be held more readily to the place; not being half so liable to slip. The second, they compact and unite the surface, and by a rotary or sliding motion, file off as it were, that portion of the gold which cannot be forced into the cavity. In the use of these instruments a continuous, straight, heavy pressure is not the most effectual; but the motion should be made so as to rock the instrument, as it were, back and forward on the gold.

Fillings inserted in the manner just described, in central cavities, where these have also been properly prepared, will not wear out by any process of mastication, short of that which will wear the teeth away.

The manner of filling all the central cavities is so near the same that a separate description is not necessary. I shall therefore only allude to a few particulars, which change to a certain extent the manner of introducing gold. Occasionally we have a cavity too small for entire block filling. When this is the case, I use a small block rolled up on the end of a strip of gold, and force this into its proper place ; then, with a sharp pointed plugger, carry in layer after layer until the cavity is well filled. It is well to have at all times a small strong spear-pointed plugger, to introduce the last folds of the gold. An instrument well adapted for the commencement of a filling, will not always be at all suitable to finish with.

I have alluded to one form of central cavity, which requires at this time a passing notice. This is where the sutures or fissures crossing the molar teeth, particularly the inferior, is the seat of disease ; and this follows these sutures to their extremes. But here they dip somewhat deeper than in their entire course ; and the disease has here made progress somewhat in proportion to that at the center. This condition of things will often make one pretty large cavity in the center, with two, three, or four smaller ones running into this, here taking an inferior molar for example, I should fill the posterior first, second the inner, third the outer, then the center, finishing on the anterior. I wish always to fill that portion of the cavity farthest from me first, because in this way I retain a perfect view of that portion yet unfilled, and can see that my blocks or folds of gold are properly arranged.

I have not alluded to other instruments than the speculum for holding the tongue in place, and keeping the tooth dry, because I have found none which would compare to this for general use. The corner of a napkin neatly folded, and thrown around the posterior molar or dens sapientia, so that the end shall be on the outside against the cheek, the larger fold then is over the tongue. A pledget or two of cotton may still be forced in around the tooth to be filled, this will absorb any saliva, which might otherwise reach the cavity. This, however, is done after the speculum is placed over the napkin. A description of this instrument is unnecessary, for I believe it is generally used. It is held in place by the patient, and if properly constructed, and placed in the mouth so as to produce no pain when pressed upon, is of incalculable service. It is, however, only applicable to the teeth of the maxillæ. The same difficulty is not generally experienced in keeping the superior teeth free from moisture, during the operation of filling, yet even here we sometimes meet with difficulty.

Some place a large piece of cotton on the labial surface of the teeth ; hold this in place with the forefinger of the left hand ; with the thumb resting against the palatal surface of the tooth ; or reverse this order of thing when applied to the teeth of the right side. Others use a cheek holder made of pearl. This at times may answer a good purpose, yet it keeps the left hand too far from the teeth, so much so that it can be of no service in handling the gold. The corner of a napkin used as the piece of cotton, I have generally found sufficient, and full as convenient. Custom in this as well as in almost every thing, will generally give success and choice of the method used. In the upper teeth, as we advance back in the mouth, difficulty of access and consequently of filling increases. The manner of preparing the gold, of introducing and compressing, has been described. This is applicable to the use of gold ; also tin when used in blocks. But I have generally found this metal in foil to possess so little adhesiveness, that the folds will not well adhere together ; hence, when using this article other than in blocks, I have cut in broad strips and rolled in cylindrical form. Of late years, however, it is so little used that I have taken no special pains to test the best method of using it, unless, indeed, a few large fillings inserted in the form of blocks ; these soon acquired a solidity of surface, and bore a better finish than I have usually expected from this metal. No amount of labor, however, can make it as hard as gold. After the filling has been introduced and consolidated, the projecting foil must be cut to the proper level, and thus prepared for a proper finish. This is done with files and scraping instruments, so constructed as to accomplish this object with very little trouble. The perfection to which files have been made for this purpose, the warmest gratitude of the profession is due Mr. Murphy, the manufacturer, as well as those of our brethren, who have taken the pains to form, and labor to supply him with the requisite patterns. With these instruments the plug is cut down level with the margin of the cavity; then with slightly blunted pluggers, pressure is made around the center border of the filling. The edges trimmed of all roughness. The plug must be trimmed so that the antagonizing tooth will not strike upon it. [*Dent. Reg. of the West.*

